# Noncovalent hyaluronan crosslinking by TSG-6: Modulation by heparin, heparan sulfate, and PRG4

**DOI:** 10.3389/fmolb.2022.990861

**Published:** 2022-10-05

**Authors:** Yun Jin Ashley Sin, Rebecca MacLeod, Adam P. Tanguay, Andrew Wang, Olivia Braender-Carr, Teraesa M. Vitelli, Gregory D. Jay, Tannin A. Schmidt, Mary K. Cowman

**Affiliations:** ^1^ Department of Biomedical Engineering, Tandon School of Engineering, New York University, New York, NY, United States; ^2^ Department of Biomedical Engineering, School of Dental Medicine, UConn Health, Farmington, CT, United States; ^3^ New York Medical College, Valhalla, NY, United States; ^4^ Department of Emergency Medicine, Warren Alpert Medical School and School of Engineering, Brown University, Providence, RI, United States; ^5^ Department of Orthopedic Surgery, Grossman School of Medicine, New York University, New York, NY, United States

**Keywords:** hyaluronan, TSG-6, PRG4, lubricin, heparin, heparan sulfate, versican, glycosaminoglycan

## Abstract

The size, conformation, and organization of the glycosaminoglycan hyaluronan (HA) affect its interactions with soluble and cell surface-bound proteins. HA that is induced to form stable networks has unique biological properties relative to unmodified soluble HA. AlphaLISA assay technology offers a facile and general experimental approach to assay protein-mediated networking of HA in solution. Connections formed between two end-biotinylated 50 kDa HA (bHA) chains can be detected by signal arising from streptavidin-coated donor and acceptor beads being brought into close proximity when the bHA chains are bridged by proteins. We observed that incubation of bHA with the protein TSG-6 (tumor necrosis factor alpha stimulated gene/protein 6, TNFAIP/TSG-6) leads to dimerization or higher order multimerization of HA chains in solution. We compared two different heparin (HP) samples and two heparan sulfate (HS) samples for the ability to disrupt HA crosslinking by TSG-6. Both HP samples had approximately three sulfates per disaccharide, and both were effective in inhibiting HA crosslinking by TSG-6. HS with a relatively high degree of sulfation (1.75 per disaccharide) also inhibited TSG-6 mediated HA networking, while HS with a lower degree of sulfation (0.75 per disaccharide) was less effective. We further identified Proteoglycan 4 (PRG4, lubricin) as a TSG-6 ligand, and found it to inhibit TSG-6-mediated HA crosslinking. The effects of HP, HS, and PRG4 on HA crosslinking by TSG-6 were shown to be due to HP/HS/PRG4 inhibition of HA binding to the Link domain of TSG-6. Using the AlphaLISA platform, we also tested other HA-binding proteins for ability to create HA networks. The G1 domain of versican (VG1) effectively networked bHA in solution but required a higher concentration than TSG-6. Cartilage link protein (HAPLN1) and the HA binding protein segment of aggrecan (HABP, G1-IGD-G2) showed only low and variable magnitude HA networking effects. This study unambiguously demonstrates HA crosslinking in solution by TSG-6 and VG1 proteins, and establishes PRG4, HP and highly sulfated HS as modulators of TSG-6 mediated HA crosslinking.

## Introduction

The glycosaminoglycan hyaluronan (HA) holds a unique place in the organization of the pericellular matrix (PCM) (Evanko et al., 2007; Knudson et al., 2018). HA is synthesized by integral membrane synthase enzymes, is extruded directly into the pericellular space, and becomes tethered to the receptor protein CD44 (Yang et al., 2012; Qu et al., 2014; Vigetti et al., 2014; Jokela et al., 2015; Kobayashi et al., 2020). The covalent structure of HA is a homogeneous repeating disaccharide polymer of [(1→4)-β-D-GlcA-(1→3)-β-D-GlcNAc] (Meyer, 1958) with a very high molecular weight of about 1–6 MDa in most healthy tissues (Cowman et al., 2015a). Each HA chain adopts an expanded wormlike coil conformation with a large hydrodynamic domain and can provide a scaffold for organization of matrix proteins and proteoglycans (Kimura et al., 1979; Morgelin et al., 1988; Cowman and Matsuoka, 2005; Cowman et al., 2015b). The localization of HA at the cell surface is closely related to the density, clustering, and state of activation of the CD44 receptors, as well as the interaction of the CD44 cytoplasmic tails with the cortical actin skeleton (Lesley and Hyman, 1998; Lesley et al., 2000; Jokela et al., 2015). The HA-CD44-actin linkage has been shown to resemble a “picket fence” arrangement, in which CD44 pickets link the intracellular actin cytoskeleton with the extracellular HA network (Kusumi et al., 2012; Freeman et al., 2018). As the extracellular component of the picket fence, HA contributes to the modulation of membrane organization and fluidity. During inflammation, the HA matrix can be weakened or disrupted by hyaluronidases or by degradation due to reactive oxygen and nitrogen species (Li et al., 1997; Stern et al., 2007; Yoshida et al., 2013; Cowman, 2017; Yamamoto et al., 2017; Yamaguchi et al., 2019; Kobayashi et al., 2020; Yoshida et al., 2020). Compensatory crosslinking of HA is a potential mechanism for rescue of matrix stiffness, membrane organization, and control of receptor signaling activity.

The secreted protein TSG-6 (tumor necrosis factor alpha stimulated gene/protein 6, TNFAIP/TSG-6) may play a physiologically important role in crosslinking HA during inflammation (Wisniewski and Vilcek, 1997; Milner and Day, 2003; Milner et al., 2006; Day and Milner, 2019). TSG-6 was discovered as the product of one of eight genes induced in human fibroblasts by TNF-α (Lee et al., 1990). Partial sequencing of the cDNA for TSG-6 showed significant similarity with the HA-binding Link domain of CD44 (Lee et al., 1990). The full amino acid sequence (Lee et al., 1992) showed a mature secreted protein of 260 amino acids with two globular domains–an approximately 94 amino acid Link domain, and an approximately 113 amino acid CUB domain–and additional N- and C- terminal peptides. The folded structures of the Link and CUB domains of TSG-6 have recently been predicted by the AlphaFold AI system (https://alphafold.ebi.ac.uk/entry/P98066) (Deep Mind and EMBL-EBI) (Jumper et al., 2021; Varadi et al., 2022) and are closely similar to the structures of the individual domains as determined from NMR and X-ray diffraction experiments and modeling based on the experimentally-derived constraints (Kohda et al., 1996; Briggs et al., 2015). The N- and C-terminal peptides appear to be intrinsically disordered, and the functions and interactions of those peptides are not known.

Binding of full length TSG-6 to HA was first shown by its co-precipitation with HA using cetyl pyridinium chloride and by its binding to HA-Sepharose (Lee et al., 1992). The mode of HA binding has been modeled from the experimentally determined structure of recombinant Link_TSG6 domain (Kahmann et al., 2000; Blundell et al., 2003; Blundell et al., 2005; Higman et al., 2007; Higman et al., 2014). The Link module forms an HA-binding groove, in which CH-π stacking with aromatic amino acids and salt bridges stabilize the complex. Link_TSG6 also binds chondroitin 4-sulfate (C4S), but not chondroitin 6-sulfate (C6S), at the HA-binding site (Parkar and Day, 1997; Park et al., 2016). Heparin (HP) binds Link_TSG6, at a distinct site from HA, but HA and HP cannot bind simultaneously (Higman et al., 2007; Park et al., 2016). Full length TSG-6 differs from the Link module in binding specificity (C6S can bind full length TSG-6) and in the increased affinity between full length TSG-6 and HA, C4S, C6S, and HP at neutral pH (Wisniewski et al., 2005).

There are two proposed mechanisms by which TSG-6 can crosslink HA. The best characterized mechanism is TSG-6-mediated catalysis of the covalent transfer of heavy chain (HC) domains from Inter-α-Inhibitor (IαI) to HA (Huang et al., 1993; Wisniewski et al., 1994; Rugg et al., 2005; Sanggaard et al., 2005; Sanggaard et al., 2006; Colon et al., 2009; Sanggaard et al., 2010). This creates HC-modified HA, which becomes crosslinked by noncovalent HC-HC self-association (Yingsung et al., 2003; Zhuo et al., 2004; Zhuo et al., 2006). The second mechanism is purely noncovalent, based on TSG-6 binding to HA, and TSG-6 dimerization serving to bring HA chains together. Evidence for the second mechanism relies on several observations. Full length TSG-6 self-associates in solution (Kim et al., 2016). HA-TSG-6 complexes show higher avidity than HA alone in binding to CD44 on lymphoid cells (Lesley et al., 2004) and in binding LYVE-1 on lymphatic endothelial cells (Lawrance et al., 2016). In addition, CHO cells producing and secreting recombinant TSG-6 become aggregated, and this effect can be abrogated by reducing HA synthesis or by the addition of HP (Kim et al., 2016). In model studies, full length TSG-6 condenses a brush-like layer of surface-bound HA (Baranova et al., 2011). The binding is cooperative, suggesting multimerization of TSG6. The Link domain of TSG-6 (termed Link_TSG6) is much less effective, and not cooperatively bound, suggesting that the CUB domain participates in TSG-6 self-association.

Our study was undertaken to unambiguously document noncovalent protein-mediated HA-HA association in solution, and its modulation by competitors. We employed an AlphaLISA bead-based assay in which end-biotinylated HA chains are bound to separate streptavidin-coated donor and acceptor beads, resulting in a signal only when the beads are brought into close association by HA-HA links. TSG-6 was shown to crosslink HA in this assay. In a survey of other HA-binding proteins, versican core protein G1 domain (VG1) was also observed to crosslink HA, in agreement with previous reports (Murasawa et al., 2013; Merrilees et al., 2016), but the cartilage link protein HAPLN1 and the HA binding protein segment of aggrecan (HABP; G1-IGD-G2) showed only low and variable signal for HA crosslinking. We further tested TSG-6-mediated HA crosslinking in solution for modulation by HP and heparan sulfate (HS). In addition, we explored the TSG-6 interactome and discovered TSG-6 binding to the mucin-like glycoprotein Proteoglycan 4 (PRG4, lubricin). PRG4 was shown to disrupt HA binding to, and crosslinking by, TSG-6.

## Materials and methods

### Materials

Biotin end-labeled 50 kDa hyaluronan (bHA; HYA-B50-200507), 50 kDa HA (Hya-50-KEF-1), and 150 kDa HA (160K-0504) were obtained from Hyalose LLC or through Echelon Biosciences Inc. Recombinant human proteins TSG-6 (Trp18-Leu277; #2104-TS), HAPLN1 (Asp16-Asn 354; #2608-HP), and HABP (Aggrecan G1-IGD-G2 domain, Val20-Gly675; #1220-PG), with C-terminal 10-His tags, expressed in mouse myeloma cells, were purchased from R&D Systems Inc. Recombinant human VG1 (#G-HA01, expressed in *E. Coli*, no tag) was obtained from Echelon Biosciences. Full length recombinant human PRG4 was provided by Lµbris BioPharma (FL, United States), expressed in CHO cells and purified as described previously (Iqbal et al., 2016). Some PRG4 was biotinylated (bPRG4) using a commercially available kit (EZ-Link Sulfo-NHS-LC-Biotinylation Kit, ThermoScientific), as per the manufacturer’s instructions. Heparin 15.7 kDa (#Hep-HG-1000) and heparin 22.2 kDa (Hep-Poly-6), each having approximately 1 N-sulfate and two O-sulfate per disaccharide, were purchased from Iduron through Galen Laboratory Supplies. Heparan sulfate fraction I, 40 kDa, averaging approximately 0.40 N-sulfate and 0.35 O-sulfate per disaccharide (GAG HSI) and heparan sulfate fraction III, 9 kDa, averaging approximately 0.65 N-sulfate and 1.10 O-sulfate per disaccharide (GAG HSIII) were purchased from Iduron through Galen Laboratory Supplies. PBS (Phosphate Buffered Saline) (#P-3813) and PBS-T (PBS containing 0.05% Tween-20) (#P-3563) were from Sigma Aldrich. AlphaScreen streptavidin-coated donor beads (#6760002), AlphaLISA streptavidin-coated acceptor beads (#AL125C), AlphaScreen Histidine (Nickel Chelate) Detection Kit containing streptavidin-coated donor beads and nickel chelate acceptor beads (#6760619C), and half-area 96 well white microplates (#6002299) were purchased from Perkin Elmer. Plate sealers were obtained from R&D Systems.

### Reagent reconstitution and storage

PBS and PBS-T were dissolved in deionized water, filtered using a Corning 1 L filter system with a 0.22 μm PES filter (#430769), and stored at 4°C. 50kDa biotinylated HA (bHA) was dissolved in 0.2 µm filtered deionized water at a concentration of 2 µM (100 μg/ml) and stored at 4°C. Aliquots of bHA stock solution were mixed by gentle repeated pipetting before use, and an appropriate volume was further diluted with filtered PBS-T immediately prior to use, to a working concentration of 4 nM (200 ng/ml) for crosslinking experiments, or 1.6 nM (80 ng/ml) for direct binding assays. Solutions were used at room temperature. TSG-6 (35 kDa), HAPLN1 (40 kDa), and HABP (74 kDa) were each dissolved in 0.2 µm filtered PBS at a concentration of 4 µM (corresponding to weight concentrations of 140, 160, and 296 μg/ml, respectively) and stored at -20°C in 20 µL aliquots. VG1 (38 kDa) was diluted from 1 mg/ml in PBS as supplied to 150 μg/ml (4 μM) in filtered PBS, and stored at -20°C in 20 μL aliquots. Aliquots of protein stock solutions were brought to room temperature and mixed with gentle repeated pipetting before use. All subsequent dilutions to working concentrations were made immediately prior to use with filtered PBS-T. Sulfated glycosaminoglycans were dissolved in 0.2 µm filtered deionized water at a concentration of 1000 μg/ml and stored at 4 °C. Corresponding molar concentrations were 20 μM, 6.7 μM, 63 μM, 46 μM, 25 μM, and 110 μM, for 50 kDa hyaluronan (HA50K), 150 kDa hyaluronan (HA150K), 16 kDa heparin (HP16K), 22 kDa heparin (HP22K), 40 kDa heparan sulfate (HSI), and 9 kDa heparan sulfate (HSIII), respectively. All dilutions were made with filtered PBS-T, and brought to room temperature. PRG4 was supplied at 1.33 mg/ml in PBS +0.01% Tween-20, and stored at -20 °C in 50 µL aliquots. Using a molecular weight of 240 kDa, the stock solution of PRG4 had a calculated molar concentration of 5.54 μM. AlphaLISA and AlphaScreen bead suspensions were supplied at 5000 μg/ml and stored at 4°C. Bead suspensions were handled in the dark, under subdued green light, and kept protected from light during all subsequent steps. Beads were vortexed for 10 s to mix before use. For crosslinking experiments in which the donor and acceptor beads are introduced simultaneously, 2 μL donor beads and 2 μL acceptor beads were mixed with 96 μL PBS-T at room temperature, to create a working solution in which each bead type is at 100 μg/ml. For direct binding assays in which the acceptor and donor beads are added in sequential steps, each bead type was diluted to 80 μg/ml in PBS-T.

### AlphaLISA platform for detection of HA crosslinking

**FIGURE 1:**
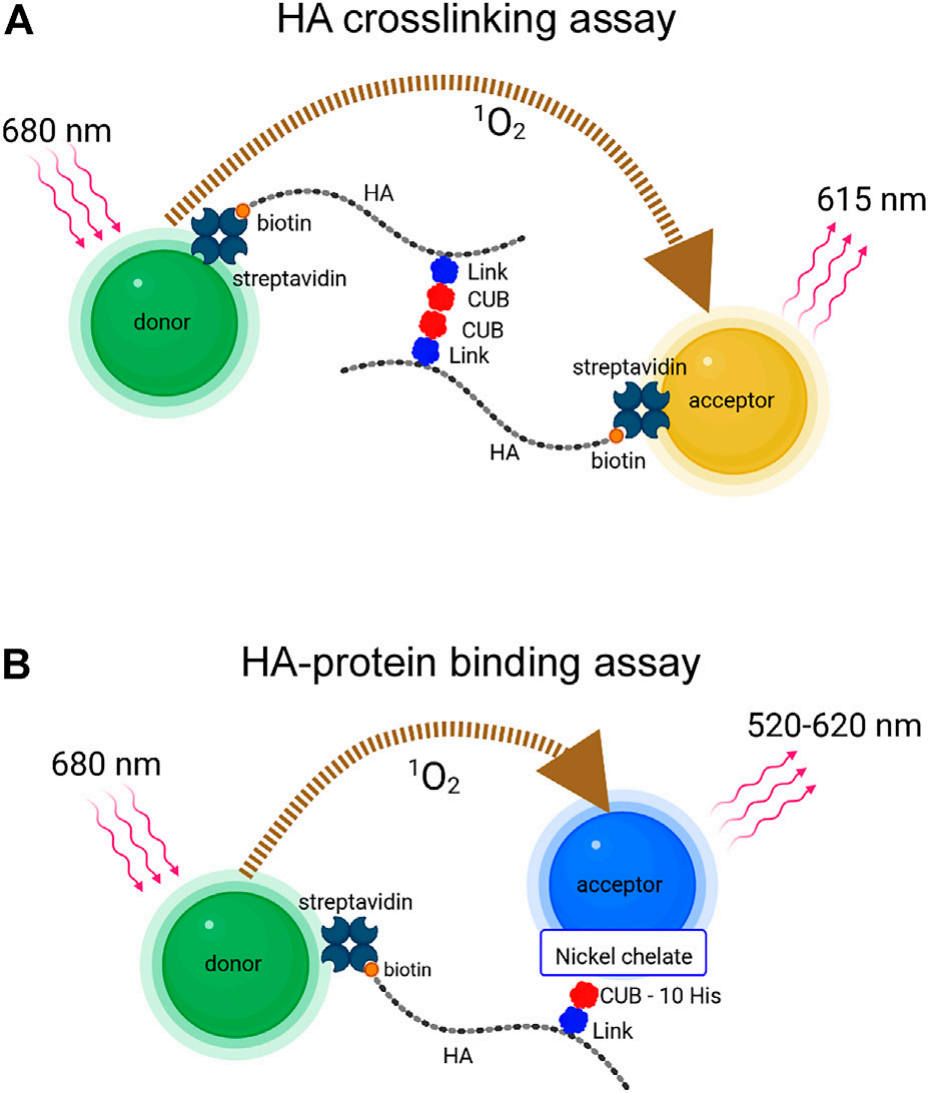
**(A)** AlphaLISA platform for detection of HA crosslinking. A protein that self-associates and simultaneously binds HA can bring together two or more end-biotinylated HA chains, allowing them to link streptavidin-coated donor (green) and acceptor (gold) beads. TSG-6 protein is illustrated as binding HA through the Link domain and self-associating through the CUB domain **(B)** AlphaScreen platform for detection of HA-protein direct binding. End-biotinylated HA binds streptavidin-coated donor beads (green). TSG-6 with a C-terminal 10-His tag binds nickel chelate-coated acceptor beads (blue). The AlphaLISA and AlphaScreen experiments involve energy transfer *via* the conversion of dissolved oxygen to excited state singlet oxygen by photosensitizer dyes in the donor beads after laser excitation, and reaction of the singlet oxygen with acceptor beads, leading to chemiluminescence. The emission wavelengths from AlphaLISA and AlphaScreen acceptor beads differ as indicated. The maximal distance for diffusion of singlet oxygen before inactivation is about 200 nm; thus donor and acceptor beads must be brought into close proximity for signal to be observed (Images were created with BioRender.com).

The assay for protein-mediated crosslinking of HA was developed using the AlphaLISA platform (Figure 1A). In this assay, streptavidin-coated donor and acceptor beads pick up nearly monodisperse 50 kDa HA (Select-HA™) molecules, which are each labeled with a single biotin at the reducing end (here called bHA). When two or more bHA chains are brought together by protein-mediated crosslinks during a pre-incubation step, donor and acceptor beads binding different bHA chains can become closely spaced. Bead proximity is detected by 680 nm laser excitation of donor bead chromophores, leading to release of singlet oxygen, and subsequent light emission at 615 nm from singlet oxygen-excited acceptor beads. Since singlet oxygen is rapidly inactivated, only bead pairs separated by less than about 200 nm can produce signal. The 50 kDa bHA chains are about 125 nm long. In our previous AlphaScreen studies of direct binding between HA and proteins on donor and acceptor beads respectively, the length of 50 kDa HA was found to provide an optimum spacing for signal, whereas HA of higher molecular weight (250–1000 kDa) gave reduced signal due to increased bead-to-bead distance (Huang et al., 2018). AlphaScreen and AlphaLISA methods differ in the identity of the chromophores inside the acceptor beads, and the choice of platform for the present studies was driven by commercial availability of acceptor beads with streptavidin coating.

### AlphaScreen platform for detection of direct HA-protein binding interactions

The assay for detection of direct binding of HA to TSG-6 was developed using the AlphaScreen platform, as previously described (Huang et al., 2018) and shown in Figure 1B. AlphaScreen acceptor beads differ from AlphaLISA beads in chromophore content and consequently in emission wavelength. For AlphaScreen experiments, excitation is at 680 nm, and broad band emission is detected from 520 to 620 nm. For our study, the donor beads carried streptavidin surface coating to bind 50 kDa bHA. The acceptor beads carried a nickel chelate coating to bind C-terminal 10-His tagged recombinant TSG-6 protein.

### Protocol for protein-mediated crosslinking of HA in solution

The AlphaLISA method for detection of HA crosslinking was previously described (Huang, 2019). bHA was used at a working concentration of 4 nM in PBS-T. Proteins were diluted to multiple working concentrations using PBS-T. In a 1.5 ml microcentrifuge tube, 32 µL PBS-T, 16 µL bHA working solution, and 16 µL protein at working concentration were mixed. The mixing order was PBS-T, bHA, then protein. Final concentrations of bHA and protein cited in each experiment were the concentrations during the overnight incubation period. Mixtures were incubated 16–22 h at 37°C with shaking at 200 rpm on a digital shaking drybath (#88880027, Thermo Fisher Scientific). The mixture of AlphaScreen donor beads and AlphaLISA acceptor beads in PBS-T at 100 μg/ml for each bead type was loaded into a microplate at 4 µL per well. Immediately after, incubated reaction mixtures were loaded into the microplate at 16 µL per well, four wells per sample. The microplate was sealed and incubated in a black box at room temperature for 1 h. The plate was read using AlphaScreen detection. Each full experiment reported was performed at least three independent times to establish reproducibility, and a representative experiment is presented.

### Protocol for competitor effects on TSG-6 mediated crosslinking of HA

bHA was used at a working concentration of 4 nM in PBS-T. TSG-6 was diluted to a working concentration of 600 nM in PBS-T. Competing agents were diluted to a series of working concentrations in PBS-T. In a 1.5 ml microcentrifuge tube, equivalent volumes of PBS-T, bHA working solution, TSG-6 working solution, and competitor at a series of working dilutions were mixed. The mixing order was PBS-T, bHA, TSG-6, then competitor. Final concentrations of bHA, TSG-6, and competitor during the overnight incubation were one-fourth the working concentrations. Mixtures were incubated 16–22 h at 37°C with shaking at 200 rpm on a digital shaking drybath. The mixture of AlphaScreen donor beads and AlphaLISA acceptor beads in PBS-T at 100 μg/ml for each bead type was loaded into a microplate at 4 µL per well. Immediately after, incubated reaction mixtures were loaded into the microplate at 16 µL per well, four wells per sample. The microplate was sealed and incubated in a black box at room temperature for 1 h. The plate was read using AlphaScreen detection. Each full experiment reported was performed at least three independent times to establish reproducibility, and a representative experiment is presented.

### Protocol for direct PRG4-TSG-6 AlphaScreen binding assay

Proteins and beads were diluted using PBS-T. bPRG4 was plated at 4x working concentrations (four times the desired final concentrations) of 0, 120 (0.500 nM), 400 (1.67 nM), 1200 (5.00 nM), 4000 (16.7 nM), 12,000 (50.0 nM), 40,000 (167 nM), and 120,000 ng/ml (500 nM). His-TSG-6 was plated at a 4x working concentration of 12,000 ng/ml (400 nM). Nickel chelate acceptor beads and streptavidin coated donor beads were each plated at a 4x working concentration of 80 μg/ml. Proteins and beads were plated at 5 μL each, for a final total volume in each well of 20 μL. Control wells lacking TSG-6 had the volume substituted with PBS-T. For order of addition, His-TSG-6 was incubated with bPRG4 for 2 h at room temperature with shaking at 200 rpm, followed by addition of nickel chelate acceptor beads for 1 h, and finally by streptavidin coated donor beads for 1 h. The plate was covered in foil during incubations and spun down after each addition of proteins or beads, as well as prior to reading, at 1000 xg for 30 s. Each combination was plated in duplicate and the plate was read using AlphaScreen detection.

### Protocol for direct HA-TSG-6 AlphaScreen binding assay and competition

bHA was diluted with PBS-T in two steps from 100 μg/ml (2 μM) to 1 μg/ml then to a working concentration of 80 ng/ml (1.6 nM), eight times the desired final concentration. HP, HS, and PRG4 were each diluted with PBS-T to a series of working concentrations, corresponding to eight times the desired final concentration. TSG-6 was diluted with PBS-T from 140 μg/ml (4 μM) to a working concentration of 2000 ng/ml (57 nM), which is four times the desired final concentration. For the initial binding step, 2.5 μL bHA, 2.5 μL competitor, and 5 μL TSG-6 were mixed in each well, and incubated at room temperature for 2 h. Then 5 μL acceptor beads at 80 μg/ml was added and incubated for 1 h. Finally, 5 μL donor beads at 80 μg/ml was added and incubated for 1 h. All final concentrations reported include the dilution factor due to addition of bead suspensions, since the pre-bead incubation duration was similar to that of the bead addition steps. The plate was read using AlphaScreen detection. Each full experiment reported was performed at least three independent times to establish reproducibility, and a representative experiment is presented.

### Instrumental methods and data analysis

AlphaLISA and AlphaScreen assay plates were read on a Molecular Devices SpectraMax i3 Multi-Mode Microplate Detection Platform using a SpectraMax AlphaScreen 384 STD Detection Cartridge (Molecular Devices, San Jose, CA). Excitation wavelength was 680 nm and emission filter was 570 nm, with 100 nm bandwidth. Excitation time was 140 ms and integration time was 280 ms. Data were collected with SoftMax Pro 7.0 from Molecular Devices. Data were analyzed and plotted (mean ± SD) using GraphPad Prism v9 software.

## Results

### Full length TSG-6 crosslinks HA in solution

In the present work, we employed a simple assay that directly detects HA crosslinking in solution to determine the effects of TSG-6 and other potential protein mediators, experimental variables, and the susceptibility of the complexes to disruption by inhibiting agents.

**FIGURE 2:**
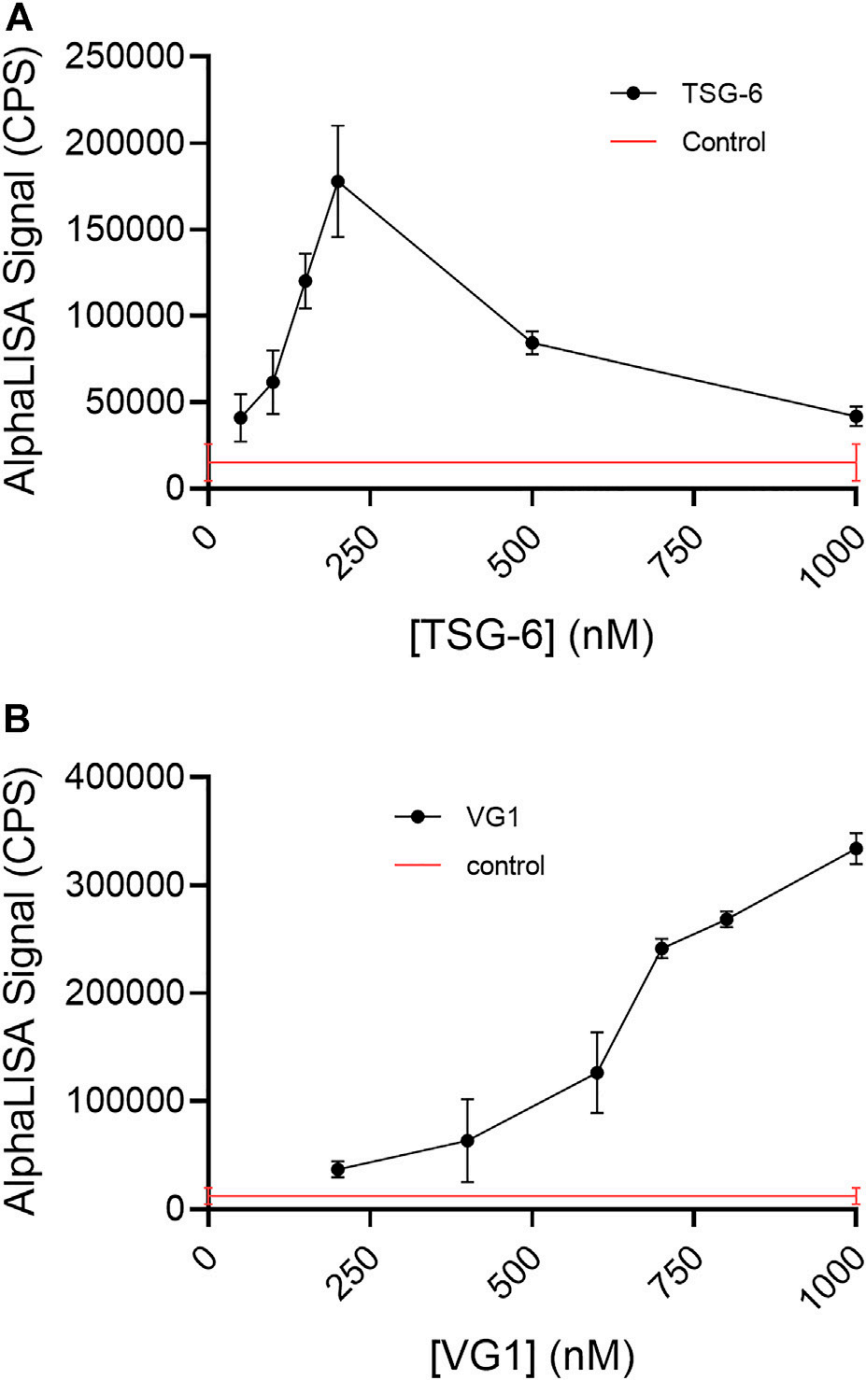
**(A)** TSG-6 and **(B)** VG1 are able to crosslink 50 kDa bHA in solution, as detected by AlphaLISA assay. For both proteins, the detectable crosslinking of bHA is dependent on the protein concentration. In the AlphaLISA experiments, end-biotinylated HA at 1 nM final chain concentration was incubated together with increasing concentrations of protein in free solution, then mixed with streptavidin-coated donor and acceptor beads for detection of HA crosslinking. The control solution contained bHA and beads only ([protein] = 0), and is shown as a line to better indicate the associated standard deviation. Signal was recorded as counts per second (CPS). Each experiment was repeated at least three independent times, and a representative experiment is shown. Each data point represents the mean ± standard deviation from quadruplicate wells.

Incubation of TSG-6 with 50 kDa bHA for 16–22 h at 37°C in PBS-T allowed both TSG-6 self-association and TSG-6 binding to HA, creating protein-mediated crosslinks. Here, crosslinked HA was detected by the addition of streptavidin-coated donor and acceptor beads, followed by AlphaLISA detection of chemiluminescent signal (measured in counts per second, CPS) when the beads are brought into close proximity (i.e., less than about 200 nm apart). bHA alone gives a very low control signal, but bHA in the presence of TSG-6 gives a high signal (Figure 2A). This is an unequivocal demonstration of TSG-6-mediated HA crosslinking in solution. The effect was found to be dependent on TSG-6 concentration. For incubation of HA at a final concentration of 1 nM (50 ng/ml) with TSG-6 at final concentrations up to about 200 nM (7 μg/ml), corresponding to a TSG-6:HA weight ratio of 140:1, the signal increased with concentration of TSG-6. Above that concentration, the signal decreased. The decrease in signal may be due to excess TSG-6 participating in CUB-CUB self-association interactions with TSG-6-bound HA (“piling on”), without linking two HA chains. Another possibility is that high TSG-6 concentrations may lead to networking of multiple HA chains, with inaccessible (“hidden”) or distant biotinylated chain ends that do not bring donor and acceptor beads within 200 nm.

### Versican G1 crosslinks HA in solution

We detected VG1-mediated HA crosslinking in solution, using the AlphaLISA assay platform (Figure 2B). Relative to the effect seen with TSG-6, a significantly higher protein concentration was needed to detect HA crosslinking. Over the concentration range studied, we did not observe a maximum concentration above which crosslinking signal decreased.

### HABP and HAPLN1 did not cause significant HA crosslinking

**FIGURE 3:**
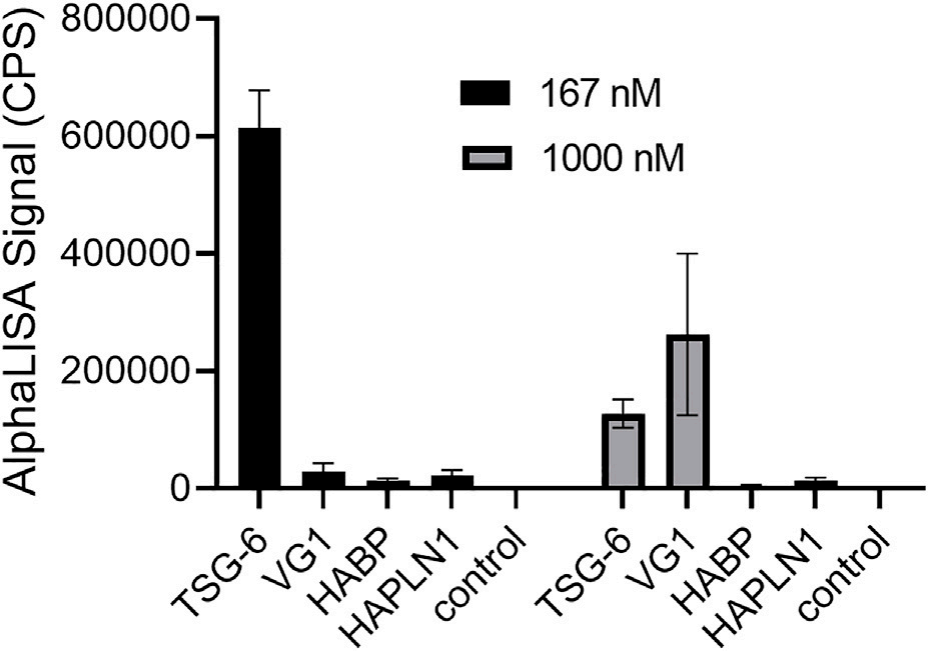
Comparison of TSG-6, VG1, aggrecan HABP, and HAPLN1 crosslinking effects on HA reveals that TSG-6 is most effective at a low concentration of 167 nM, while VG1 is more effective than TSG-6 at 1000 nM. HABP and HAPLN1 did not cause significant crosslinking of HA at either concentration. In the AlphaLISA experiments, end-biotinylated HA at 1 nM final chain concentration was incubated together with protein at the indicated concentration in free solution, then mixed with streptavidin-coated donor and acceptor beads for detection of HA crosslinking. The control solution contained bHA and beads only ([protein] = 0). Signal was recorded as counts per second (CPS). Each experiment was repeated at least three independent times, and a representative experiment is shown. Each data point represents the mean ± standard deviation from quadruplicate wells.

For the HABP (G1-IGD-G2) portion of aggrecan, we did not observe significant HA crosslinking using the AlphaLISA assay. For the cartilage link protein HAPLN1, which is known to self-aggregate, we observed a very low signal for protein-induced HA crosslinking and failed to achieve reproducible significant difference from controls in repeated experiments. (Figure 3). Strong binding of HABP and HAPLN1 to HA still occur under the conditions used, and have been previously studied and exploited in development of a sensitive and specific competitive AlphaScreen assay for HA (Huang et al., 2018). Protein binding to HA, in the absence of protein self-association in a manner that can link two end-biotinylated HA chains together (for example, side-by-side association on a single HA polymer) is not sufficient for the observation of HA crosslinking using streptavidin-coated donor and acceptor beads in the AlphaLISA assay.

### Heparin (HP) and highly sulfated heparan sulfate (HS) inhibit HA crosslinking by TSG-6

**FIGURE 4:**
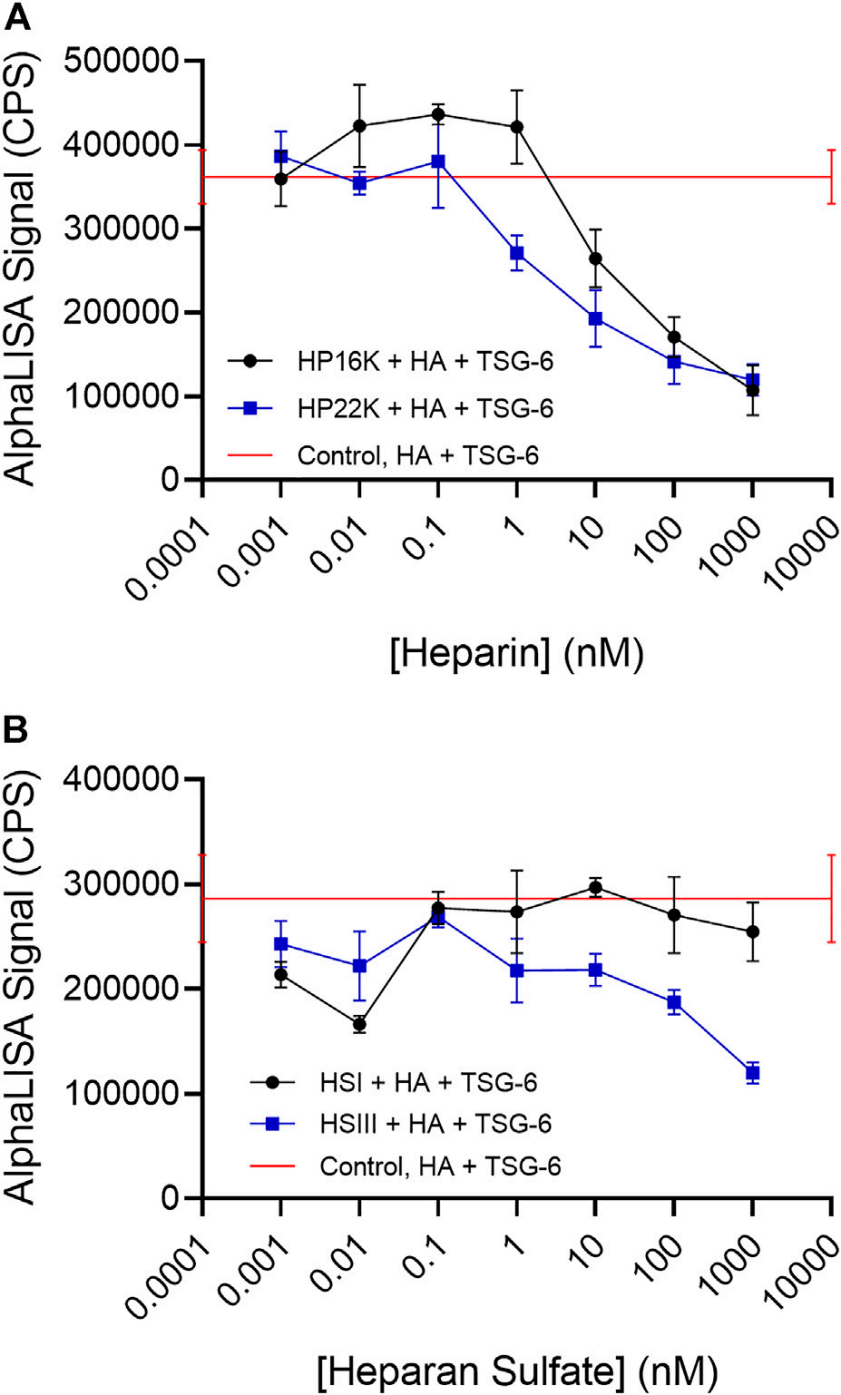
**(A)** Heparin and **(B)** Heparan sulfate can disrupt TSG-6-mediated HA crosslinking. Heparan sulfate with a low degree of sulfation (HS-I) is less effective than highly sulfated heparan sulfate (HS-III). In the AlphaLISA experiments, end-biotinylated HA at 1 nM final chain concentration was incubated together with TSG-6 protein at 150 nM final concentration, and increasing concentrations of heparin (HP) or heparan sulfate (HS) in free solution, then mixed with streptavidin-coated donor and acceptor beads for detection of HA crosslinking. The control solution contained bHA, TSG-6, and beads only ([HP] or [HS] = 0), and is shown as a line to better indicate the associated standard deviation. Signal was recorded as counts per second (CPS). Each experiment was repeated at least three independent times, and a representative experiment is shown. Each data point represents the mean ± standard deviation from quadruplicate wells.

In these studies, the bHA concentration was kept at 1 nM and the TSG-6 concentration was kept at 150 nM (below the concentration at which increased TSG-6 self-association causes the AlphaLISA signal for HA crosslinking to drop). Inhibitors were added at increasing concentrations as shown in the figures. All components were mixed together and incubated at 37°C overnight, followed by bead binding for the AlphaLISA assay. HP samples with average molecular weights of 16 kDa and 22 kDa, each having about three sulfates per disaccharide, were found to effectively inhibit TSG-6-mediated HA crosslinking (Figure 4A). The signal was reduced to about half by HP at concentrations of 10–100 nM.

We frequently observed a small increase in crosslinking signal in the presence of either HP sample at low concentrations. The cause is not certain, but HP binding to the CUB domain might potentially enhance TSG-6 self-association. That effect would be irrelevant at higher HP concentrations, when the Link domain interaction with HA is disrupted.

HS samples were more variable in effect, depending on the molecular weight and degree of sulfation (Figure 4B). A relatively highly sulfated HS fraction (HSIII) with low molecular weight (1.75 sulfates per disaccharide, 9 kDa) was able to disrupt HA crosslinking by TSG-6, but required a 10 to 100-fold higher concentration than HP to reduce the crosslinking signal by half. A lower sulfated, higher molecular weight HS (0.75 sulfate per disaccharide, 40 kDa) (HSI) showed a more variable and weaker ability to inhibit TSG-6-mediated HA crosslinking within the concentration range investigated.

### PRG4 binds TSG-6 and effectively disrupts TSG-6-mediated HA crosslinking

**FIGURE 5:**
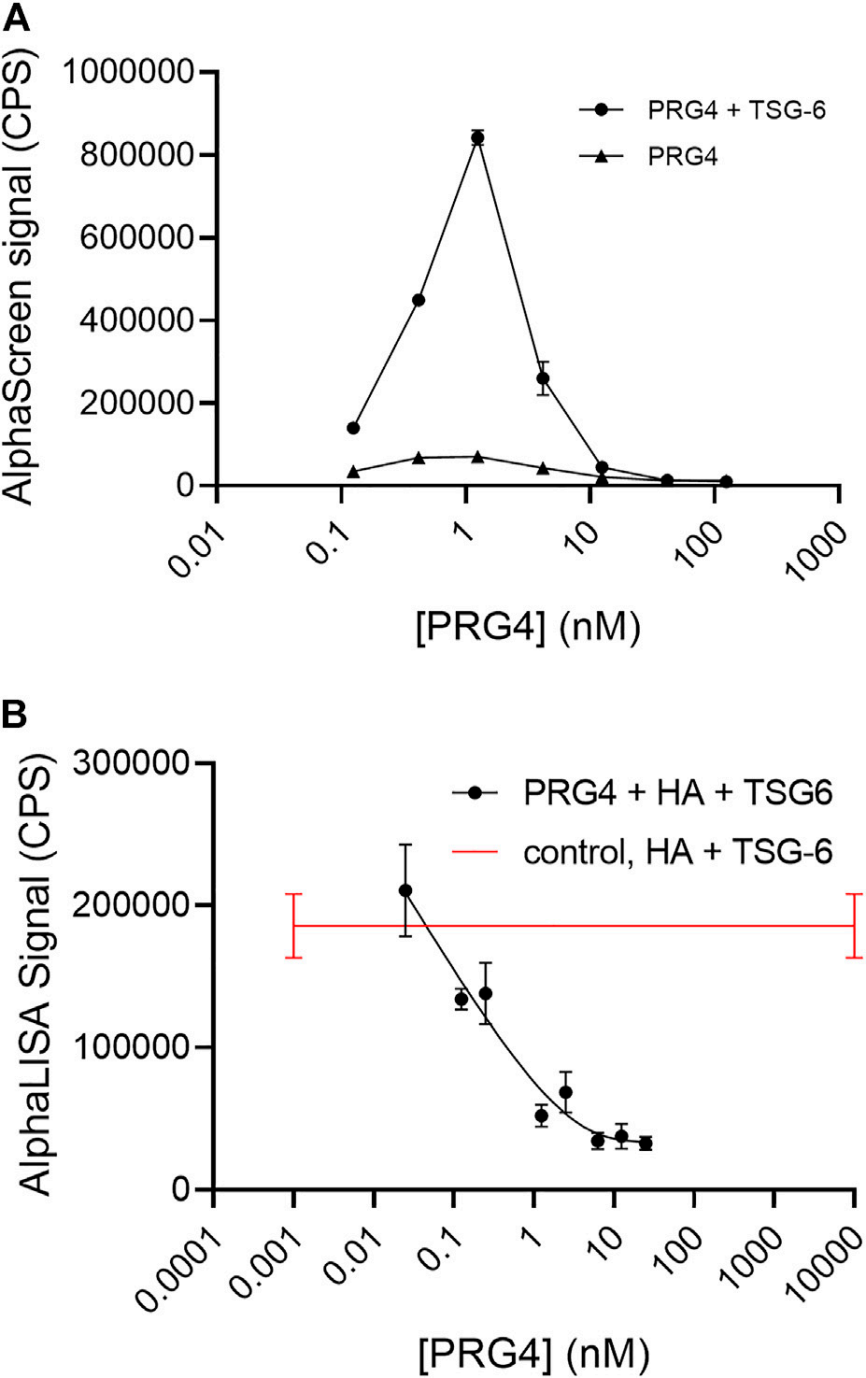
**(A)** AlphaScreen assay shows direct binding of biotinylated Proteoglycan 4 (PRG4) to His-tagged TSG-6, using streptavidin-coated donor beads and nickel chelate-coated acceptor beads. The final TSG-6 concentration was 100 nM, and the control solution lacked TSG-6. Each data point represents the mean from duplicate wells **(B)** AlphaLISA assay shows PRG4 disrupts TSG-6-mediated HA crosslinking. In the AlphaLISA experiments, end-biotinylated HA at 1 nM final chain concentration was incubated together with TSG-6 protein at 150 nM final concentration, and increasing concentrations of PRG4 in free solution, then mixed with streptavidin-coated donor and acceptor beads for detection of HA crosslinking. The control solution contained bHA, TSG-6, and beads only ([PRG4] = 0), and is shown as a line to better indicate the associated standard deviation. Signal was recorded as counts per second (CPS). Each experiment was repeated at least three independent times, and a representative experiment is shown. Each data point represents the mean ± standard deviation from quadruplicate wells.

Considering the likely importance of HA crosslinking by TSG-6 to pericellular biomechanical properties, we investigated the potential of PRG4 to bind TSG-6. In a direct binding assay performed using AlphaScreen, with biotinylated PRG4 on streptavidin donor beads and His-tagged TSG-6 on nickel chelate coated acceptor beads, we observed a strong signal for binding (Figure 5A).

PRG4 was highly effective as an inhibitor of HA crosslinking by TSG-6 (Figure 5B). At a final concentration of about 1 nM, equal to the bHA concentration, the signal due to TSG-6-mediated HA crosslinking is reduced by half. PRG4 was more effective than HP or HS at similar molar concentrations.

### Inhibition of TSG-6-mediated HA crosslinking by HP, HS, and PRG4 results from reduced HA binding to TSG-6, not reduced TSG-6 self-association

HP, HS, and PRG4 are all able to bind TSG-6. In the case of HP, the major binding site is known to be on the Link module. HS presumably binds to the same site. The binding site for PRG4 is not yet known. Disruption of HA crosslinks formed by TSG-6 self-association and binding to HA could in principle occur by inhibiting either the HA-TSG-6 binding interaction or TSG-6—TSG-6 association. In order to test whether direct HA-TSG-6 binding was disrupted by HP, HS, and/or PRG4, we used the AlphaScreen assay platform (Figure 1B). We optimized the TSG-6 concentration to a value (14 nM, 500 ng/ml) that was below the concentration at which signal saturated due to maximal loading of the nickel chelate beads. The optimum bHA concentration was previously determined to be 0.2 nM (10 ng/ml). For the binding assay, biotinylated HA, His-tagged TSG-6, and inhibitor were pre-incubated in solution, after which the TSG-6 was bound to nickel chelate coated acceptor beads and bHA by streptavidin-coated donor beads. In the absence of an inhibitor, a strong emission signal was observed. As a control experiment, unlabeled HA of 150 kDa was used as a competitor. The signal was reduced by half at a concentration of about 0.1 nM for HA-150K, close to the concentration of bHA in the solution. Unlabeled HA was an effective competitor for bHA, both when all reactants were premixed and incubated in solution before binding beads and when bHA and TSG-6 were preloaded on their respective beads. In contrast to unlabeled HA, HP and HS could not compete with bHA for TSG-6 binding if the beads were preloaded, a process that facilitates HA-TSG-6 binding. The inability of heparin to disrupt HA binding to the Link_TSG6 module unless heparin is added first was reported previously (Mahoney et al., 2005)

**FIGURE 6:**
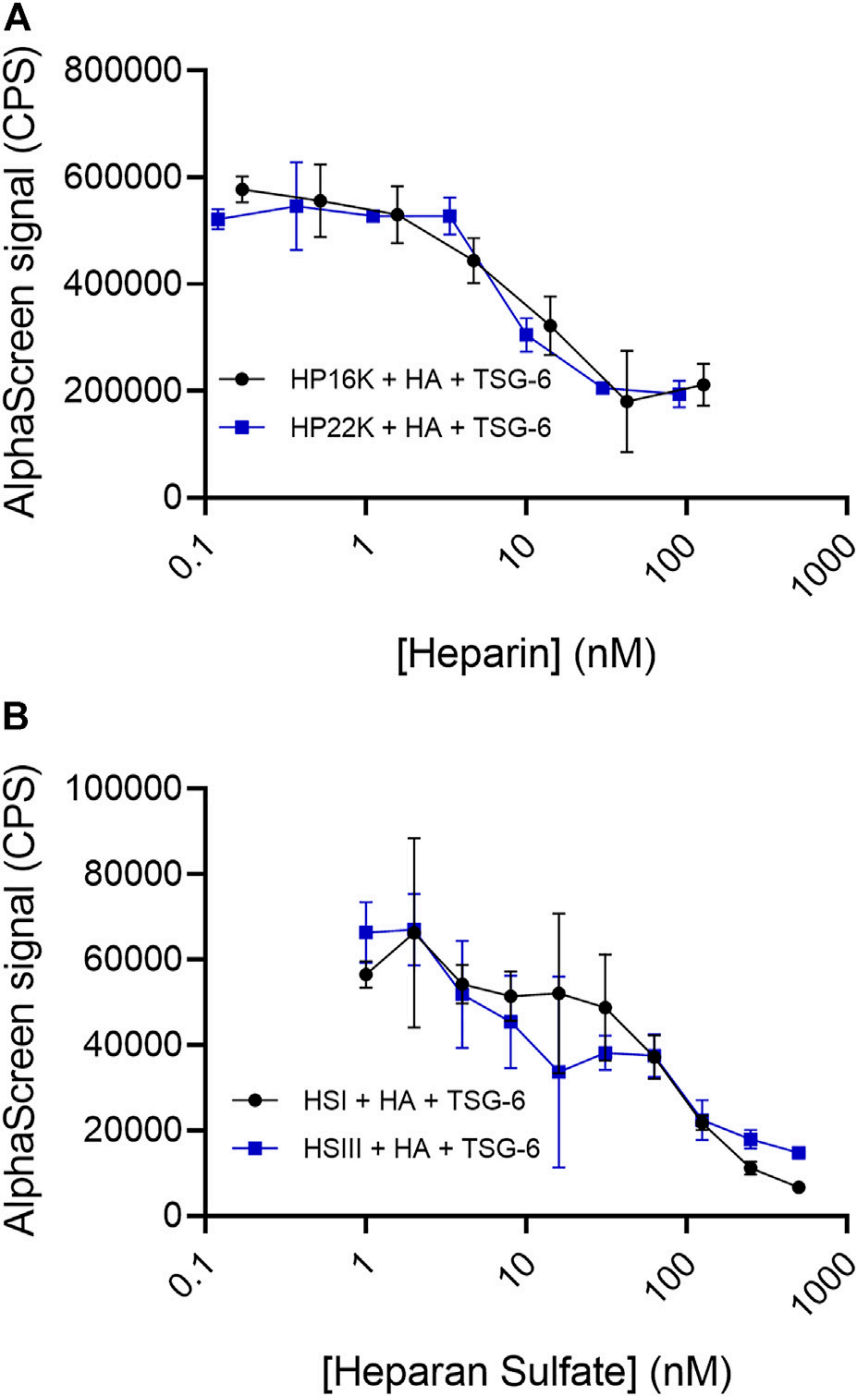
**(A)** Heparin and **(B)** Heparan sulfate compete with HA for binding to TSG-6. In the AlphaScreen experiments, end-biotinylated HA at 0.2 nM final chain concentration was incubated together with TSG-6 protein at 14 nM final concentration, and increasing concentrations of heparin (HP) or heparan sulfate (HS) in free solution, then mixed with streptavidin-coated donor and nickel chelate-coated acceptor beads for detection of HA-TSG-6 direct binding. Signal was recorded as counts per second (CPS). Each experiment was repeated at least three independent times, and a representative experiment is shown. Each data point represents the mean ± standard deviation from triplicate wells.

Using the protocol in which bHA, TSG-6, and competitor were premixed and incubated together in solution, we first examined HP and HS as competitors. HP of 16 kDa and 22 kDa molecular weight each effectively disrupted HA-TSG-6 binding (Figure 6A). HP nearly halved the signal at about 10 nM, which is a 50 times greater concentration than the 0.2 nM HA. The highly sulfated HSIII was a somewhat less effective competitor, reducing the signal by half at a concentration of about 50 nM (Figure 6B). Surprisingly, the less sulfated HSI sample was also able to disrupt direct HA-TSG-6 binding at a similar concentration. It is not yet clear why the HSI sample could disrupt direct binding of HA to TSG-6 but was less effective in disrupting TSG-6-mediated HA crosslinking over the concentration ranges tested.

**FIGURE 7:**
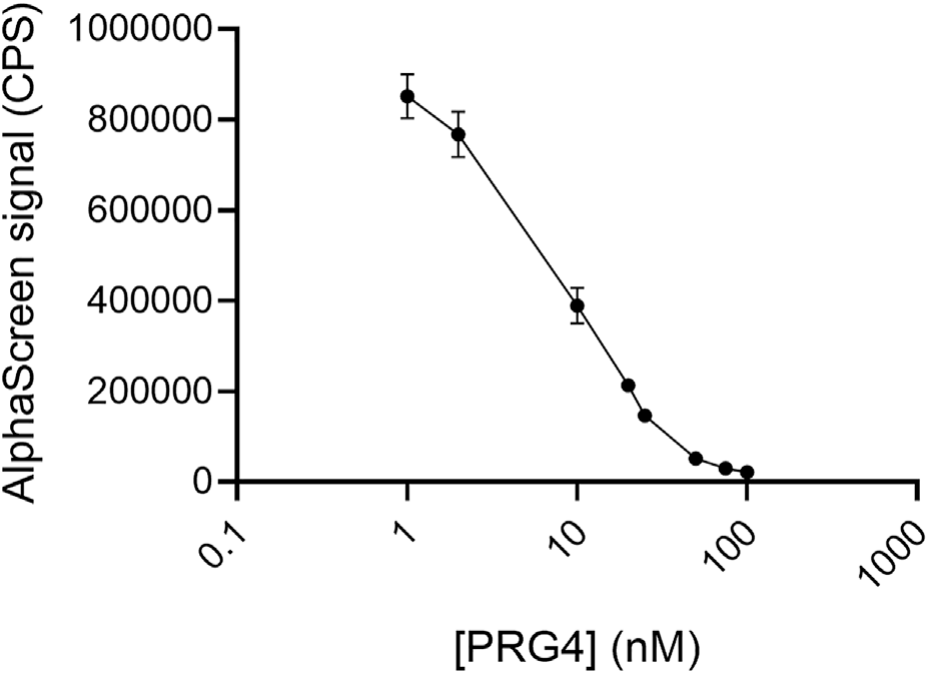
PRG4 competes with HA for binding to TSG-6. In the AlphaScreen experiments, end-biotinylated HA at 0.2 nM final chain concentration was incubated together with TSG-6 protein at 14 nM final concentration, and increasing concentrations of PRG4 in free solution, then mixed with streptavidin-coated donor and nickel chelate-coated acceptor beads for detection of HA-TSG-6 direct binding. Signal was recorded as counts per second (CPS). Each experiment was repeated at least three independent times, and a representative experiment is shown. Each data point represents the mean ± standard deviation from triplicate wells.

PRG4 was highly effective in reducing HA binding to TSG-6 (Figure 7), reducing the signal by half at a concentration of about 5–10 nM. It was similar to HP in competitive effectiveness in the direct binding assays.

## Discussion

### TSG-6 noncovalently crosslinks HA chains in solution

Noncovalent binding of the TSG-6 Link module to HA, accompanied by dimerization of TSG-6 through the CUB domain, has been previously proposed to crosslink HA (Lesley et al., 2004; Baranova et al., 2011; Kim et al., 2016; Lawrance et al., 2016). In order to confirm and provide a direct measure of TSG-6-mediated HA-HA crosslinking, we employed an AlphaLISA-based assay in which crosslinking of two end-biotinylated HA chains could be detected as a signal when streptavidin-coated donor and acceptor beads were brought into close proximity by the two HA chains. In the absence of a crosslinking protein, no signal was observed. Incubation of the HA with TSG-6 prior to bead addition gave a strong signal due to HA crosslinking. The ratio of TSG-6 to HA dictated the extent of detectable HA crosslinking, such that a large excess of TSG-6 allowed it to either self-aggregate without crosslinking HA, or to crosslink HA into networks with “hidden” ends or distances between accessible ends that exceeded the 200 nm limit for detection by AlphaLISA.

Our results are in accord with the findings of previous research groups, who documented a dependence of apparent crosslinking on the weight ratio of TSG-6 to HA. At a very low weight ratio of 0.22:1, no crosslinking was detected by measurement of solution viscosity (Fasanello et al., 2021). In studies of the effect of HA-TSG-6 on CD44 binding affinity, a TSG-6:HA weight ratio of 9:1 gave a significant increase in avidity, but the effect was reduced at a higher ratio of 30:1 (Lesley et al., 2004). For binding to LYVE-1, a TSG-6:HA weight ratio of 5:1 was effective in increasing avidity (Lawrance et al., 2016). In studies of TSG-6 condensation of a surface-tethered HA layer, HA condensation increases and then decreases with the addition of TSG-6 (Baranova et al., 2011).

Sensitivity of HA crosslinking to the ratio of TSG-6 to HA may also depend on the effective degree of self-association of TSG-6 prior to binding HA, and the reversibility of that self-association, which might in turn depend on sample history. Recombinant full-length TSG-6 self-association in solution has been reported to depend on thermal history (Kim et al., 2016). For our experiments, TSG-6 dissolved in PBS at a concentration of 4 μM (140 μg/ml) had been stored frozen in small aliquots until use. An extended overnight incubation time at 37°C was employed to create the complexes. A high degree of reproducibility was achieved in these experiments, but it can be anticipated that different TSG-6 samples, with different thermal or handling histories, may have different optimum concentrations for HA crosslinking. Confidence in the existence of the crosslinking effect is nonetheless derived from the consistent observations of complex formation and concentration ratio dependence, linking the present studies to prior work. The simple bead-based assay platform for HA crosslinking is thus established as a useful experimental approach for the study of protein-mediated HA crosslinking and its competition by various matrix components.

### Heparin and heparan sulfate inhibit HA crosslinking by disrupting HA binding to the link domain of TSG-6

TSG-6-mediated HA crosslinking was disrupted by HP and highly sulfated HS, while low sulfated HS was less effective. The ability of HP to disrupt or inhibit HA crosslinking by TSG-6 is in accord with expectations. The Link domain of TSG-6 has been shown to bind HP (Mahoney et al., 2005; Higman et al., 2007; Marson et al., 2009). The HP-binding site is distinct from the HA-binding site on TSG-6, and mutation of the HP binding site does not destroy HA binding (Jadin et al., 2014; Bano et al., 2018). In spite of the different binding sites, HP noncompetitively inhibits HA binding to TSG-6, possibly by favoring a conformational change that inhibits HA binding (Higman et al., 2007). HP has also been reported to disrupt TSG-6-mediated crosslinking of HA that can cause cell aggregation. (Kim et al., 2016).

The effect of HS in disrupting HA crosslinking by TSG-6 has not previously been reported. To determine whether HP and HS disrupt the HA-TSG-6 binding, or disrupt TSG-6 self-association, we tested the direct binding of HA to TSG-6 using an AlphaScreen bead-based assay. We observed that HP and HS (both high and low sulfated forms) effectively disrupt direct binding of the TSG-6 Link module to HA.

An interesting observation is that HP at low levels causes a slight increase in HA crosslinking by TSG-6 but has no similar effect on direct HA-TSG-6 binding. It may be possible that HP binding to the CUB domain (Milner and Day, 2003) can enhance TSG-6 self-association, but when HP is present at sufficiently high levels, HP disruption of the Link module binding to HA is the dominant effect.

### PRG4 binds TSG-6 and inhibits HA-TSG-6 binding and crosslinking

TSG-6 has a large number of protein binding partners in the extracellular matrix, including multiple growth factors and chemokines, as well as fibronectin, thrombospondin-1, and pentraxin-3 (Day and Milner, 2019). We found that PRG4 also binds to TSG-6 in solution and can very effectively inhibit HA binding to TSG-6, abrogating HA crosslinking. No direct binding between PRG4 and HA was observed, using bHA on streptavidin-coated donor beads and mAb 9G3-bound PRG4 on protein G acceptor beads, (data not shown). PRG4 has been shown to bind CD44 (Al-Sharif et al., 2015) (and inhibit HA stimulated signaling (Sarkar et al., 2019)), and more recently MMP9 (Menon et al., 2021) (which associates with CD44 as well (Yu and Stamenkovic, 1999)). Given PRG4’s anti-inflammatory (Qadri et al., 2018; Das et al., 2019; Menon et al., 2021; Krawetz et al., 2022), immunomodulatory (Qadri et al., 2021; Krawetz et al., 2022), and anti-fibrotic properties (Qadri et al., 2020; Krawetz et al., 2022), it is intriguing to consider what type of additive, synergistic, or inhibitory biological properties TSG-6 bound PRG4 might have in various tissues and diseases.

### VG1 noncovalently crosslinks HA chains in solution

The structure, occurrence, and binding interactions of the proteoglycan versican have been recently reviewed (Islam and Watanabe, 2020; Wight et al., 2020). Like aggrecan, the protein core has an N-terminal globular G1 domain, which binds specifically to HA. Unlike the aggrecan AG1 domain, VG1 has a marked tendency to self-associate in solution, and to bind HA cooperatively (Seyfried et al., 2006). Functionally, VG1 fragments can aggregate with the VG1 domain of full length versican, linked *via* its C-terminal G3 domain to fibrillin-1, and foster increased HA association with fibrillin-1 coated elastin fibers (Murasawa et al., 2013). Additionally, VG1 fragments produced during inflammation, but not full length versican, bind pericellular HA and its partners, TSG-6 and IαI, to form large cable-like assemblies that function in binding leukocytes (Merrilees et al., 2016). Using the AlphaLISA assay, we demonstrated that recombinant VG1 alone can mediate HA-HA crosslinking in solution. Interestingly, the HAPLN1 protein, which also self-associates in solution and binds HA cooperatively (Seyfried et al., 2005), showed only very low and variable HA crosslinking signal in our studies. We did not detect significant HA crosslinking in the presence of aggrecan HABP (G1-IGD-G2).

### A model for the role of TSG-6, HSPG, and PRG4 in modulation of HA effects on plasma membrane organization and receptor signaling

The plasma membrane of a cell is organized into spatial compartments by the underlying cortical actin filament network, linked to transmembrane proteins, acting together as a picket fence (Kusumi et al., 2012). Immobilized (confined) transmembrane proteins act as obstacles to diffusion of phospholipids and other proteins, such that diffusion within a compartment is much more rapid than diffusion between compartments. The plasma membrane compartmentalization strongly affects receptor protein dimerization and oligomerization.

In agreement with this model, CD44 acts as a picket protein, with its cytoplasmic tail bound to the actin skeleton *via* ezrin. CD44 localization and lateral diffusion have been shown to depend on the organization and dynamics of the cortical actin skeleton in macrophages, with 70–85% of CD44 being immobile or spatially confined (Freeman et al., 2018; Mylvaganam et al., 2018; Vega et al., 2018). In addition, HA bound to CD44 was found to act as a pericellular exoskeleton, further restricting the mobility of CD44 and receptor proteins (Freeman et al., 2018). In other studies, clustering of CD44 was found to be enhanced by high molecular weight HA and reduced by low molecular weight HA (Yang et al., 2012). The link between HA size and CD44 mobility suggests a connection between HA degradation during inflammation and consequent effects on mobility of receptor proteins for inflammatory cytokines and PAMPs/DAMPs. Receptor mobility in turn controls the rate of dimerization or oligomerization, thus influencing ligand affinity and activation of signaling pathways (Wong et al., 2020). High molecular weight HA is protective, and fragmented HA is more permissive for inflammatory signaling. The increased production of high molecular weight HA in response to inflammatory stimulus is a natural defense mechanism (Cowman 2017).

TSG-6 production is similarly increased as a protective response during inflammation. In this study, we found that TSG-6 self-association can crosslink HA in solution, effectively increasing the HA molecular weight. Crosslinking of pericellular HA by TSG-6 may contribute to protection against inflammatory signaling through stabilization of the outer portion of the picket fence organization of the plasma membrane and modulation of pro-inflammatory receptor diffusion, oligomerization, and signaling.

Heparan sulfate proteoglycans (HSPG) and PRG4 can inhibit the degree of HA crosslinking by TSG-6 *via* their binding interactions with TSG-6. Thus, reversible noncovalent crosslinking of HA can be modulated by the presence of HSPG and PRG4.

## Data Availability

The raw data supporting the conclusions of this article will be made available by the authors, without undue reservation.
